# Ventilation during continuous compressions or at 30:2 compression-to-ventilation ratio results in similar arterial oxygen and carbon dioxide levels in an experimental model of prolonged cardiac arrest

**DOI:** 10.1186/s40635-022-00485-0

**Published:** 2023-01-06

**Authors:** Jukka Kopra, Erik Litonius, Pirkka T. Pekkarinen, Merja Laitinen, Juho A. Heinonen, Luca Fontanelli, Tomi P. Mäkiaho, Markus B. Skrifvars

**Affiliations:** 1 Department of Emergency Care and Services, Helsinki University Hospital and University of Helsinki, Helsinki, Finland; 2 Division of Anaesthesiology, Department of Anaesthesiology, Intensive Care and Pain Medicine, University of Helsinki and Helsinki University Hospital, Helsinki, Finland; 3 Division of Intensive Care, Department of Anaesthesiology, Intensive Care and Pain Medicine, University of Helsinki and Helsinki University Hospital, Helsinki, Finland; 4 VetCT Teleconsulting–Teleradiology Small Animal Team, Cambridge, UK; 5 Department of Clinical-Surgical, Diagnostic and Paediatric Sciences, Unit of Anaesthesia and Intensive Care, University of Pavia, Pavia, Italy

**Keywords:** Cardiac arrest, Cardiopulmonary resuscitation, Ventilation, Hypoxaemia, Arterial oxygen pressure, Mechanical chest compression, Electrical impedance tomography, Computed tomography

## Abstract

**Background:**

In refractory out-of-hospital cardiac arrest, transportation to hospital with continuous chest compressions (CCC) from a chest compression device and ventilation with 100% oxygen through an advanced airway is common practice. Despite this, many patients are hypoxic and hypercapnic on arrival, possibly related to suboptimal ventilation due to the counterpressure caused by the CCC. We hypothesized that a compression/ventilation ratio of 30:2 would provide better ventilation and gas exchange compared to asynchronous CCC during prolonged experimental cardiopulmonary resuscitation (CPR).

**Methods:**

We randomized 30 anaesthetized domestic swine (weight approximately 50 kg) with electrically induced ventricular fibrillation to the CCC or 30:2 group and bag-valve ventilation with a fraction of inspired oxygen (FiO_2_) of 100%. We started CPR after a 5-min no-flow period and continued until 40 min from the induction of ventricular fibrillation. Chest compressions were performed with a Stryker Medical LUCAS® 2 mechanical chest compression device. We collected arterial blood gas samples every 5 min during the CPR, measured ventilation distribution during the CPR using electrical impedance tomography (EIT) and analysed post-mortem computed tomography (CT) scans for differences in lung aeration status.

**Results:**

The median (interquartile range [IQR]) partial pressure of oxygen (PaO_2_) at 30 min was 110 (52–117) mmHg for the 30:2 group and 70 (40–171) mmHg for the CCC group. The median (IQR) partial pressure of carbon dioxide (PaCO_2_) at 30 min was 70 (45–85) mmHg for the 30:2 group and 68 (42–84) mmHg for the CCC group. No statistically significant differences between the groups in PaO_2_ (*p* = 0.40), PaCO_2_ (*p* = 0.79), lactate (*p* = 0.37), mean arterial pressure (MAP) (*p* = 0.47) or EtCO_2_ (*p* = 0.19) analysed with a linear mixed model were found. We found a deteriorating trend in PaO_2_, EtCO_2_ and MAP and rising PaCO_2_ and lactate levels through the intervention. There were no differences between the groups in the distribution of ventilation in the EIT data or the post-mortem CT findings.

**Conclusions:**

The 30:2 and CCC protocols resulted in similar gas exchange and lung pathology in an experimental prolonged mechanical CPR model.

**Supplementary Information:**

The online version contains supplementary material available at 10.1186/s40635-022-00485-0.

## Background

In refractory out-of-hospital cardiac arrest (OHCA), the patient is commonly transported to hospital to be assessed for suitability for extracorporeal cardiopulmonary resuscitation (ECPR). During transport, mechanical chest compressions are recommended because they allow high-quality compressions in a moving vehicle and ensure rescuer safety [1, 2]. Ventilation is generally conducted with 100% oxygen through an advanced airway, such as an endotracheal intubation tube.

Despite ventilation with 100% oxygen, most patients have severe hypoxia and hypercapnia after prolonged CPR [3, 4]. One possible explanation is ventilating during the counterpressure phase of chest compressions, which results in air trapping [5, 6]. In a large patient study with manual compressions, resuscitation with a 30:2 ratio resulted in similar survival outcomes to continuous chest compressions (CCC) [7]. Although it is possible to achieve superior perfusion pressures with mechanical CPR [8], there is also evidence of more visceral trauma to the internal organs [9] including the lungs [10], in comparison with manual compressions. Cardiopulmonary resuscitation-associated lung oedema (CRALE) associated with mechanical compressions may also impact the effectiveness of ventilation during CPR [11].

It is currently unclear whether mechanical chest compressions performed either continuously or at a 30:2 ratio affect differently the performance of ventilation and oxygenation or the development of lung injury. Therefore, we designed the current experimental study in which we hypothesized that a 30:2 compression-to-ventilation ratio would provide better arterial blood oxygenation and less hypercapnia compared to CCC. In addition, we hypothesized that the 30:2 compression to ventilation protocol would result in less lung injury based on the findings of a post-mortem computed tomography (CT) scan and a more even distribution of ventilation using electrical impedance tomography (EIT) recordings conducted during CPR.

## Material and methods

We conducted this experimental animal study on healthy juvenile pigs at the Laboratory Animal Centre, Large Animals Unit at the Viikki Campus of the University of Helsinki between September 2020 and January 2021. The study was approved by the Finnish National Animal Experiment Board (ESAVI/16960/2020). The study is reported in adherence to the ARRIVE guidelines, and a checklist is included in the Additional file 1.

### Preparation and monitoring

We included 30 healthy Landrace pigs of both sexes weighing between 44 and 52 kg. On the procedural day, the animals were fasted, but could freely access water. Thirty minutes before the experiment, the animals received intramuscular injections of medetomidine (8–9 mg) and racemic ketamine (400–450 mg) as premedication. Once sedated, a peripheral ear vein was cannulated, and a crystalloid infusion (Ringer-Acetat Baxter, Baxter Medical, Kista, Sweden) was started. The pigs were anaesthetized with intravenous (IV) propofol (dose 1–1.5 mg/kg) and fentanyl (3–4 mcg/kg). Anaesthesia was maintained with propofol infusions (20 mg/ml, 5–25 ml/h) and fentanyl boluses. The pigs were intubated (endotracheal tube sizes 6.0 or 7.0) and mechanically ventilated (Servo Ventilator 900C; Siemens-Elema, Solna, Sweden) with 21% oxygen (O_2_) during the pre-arrest period. End-tidal carbon dioxide (etCO_2_) was kept at the 5% level. The internal jugular vein was accessed using Seldinger’s technique, and an introducer catheter (Arrow, size 8.5Fr. 16 cm, Teleflex Medical Europe Ltd, Westmeath, Ireland) was inserted for central venous blood sampling and pacemaker insertion. The femoral artery was prepared surgically and cannulated using an introducer sheath (Avanti+, size 6F, length 11 cm, Cordis, Tipperary, Ireland) for invasive blood pressure measurement and for obtaining arterial blood samples. After collecting the baseline blood samples, a temporary balloon-tipped pacing wire was inserted. Location in the right ventricle of the heart was confirmed with the achievement of ventricular pacing using an external pacemaker (Medtronic 5348 Single Chamber Temporary Pacemaker, Soma Tech INTL, Bloomfield, CT, USA).

Oxygen saturation (SpO_2_) was monitored with pulse oximetry from the pigs’ tails. Arterial blood gases were measured with a point-of-care device (i-STATSystem, Abbott Laboratories, Princeton, NJ, USA). Ventilation parameters were monitored using a spirometry flow sensor (D-Lite, GE Healthcare, USA) connected to an airway module, with the results visualized on a vital signs monitor (GE DATEX-OHMEDA, GE Healthcare, Helsinki, Finland). Haemodynamic and respiratory variables were monitored using an AS/3Monitor (Datex-Ohmeda AS/3, GE Healthcare, Helsinki, Finland) and recorded using data collection software (iCentral® and S/5 Collect®, GE Healthcare, Helsinki, Finland). A rectal temperature probe was inserted, and a temperature of 38 °C–39 °C was targeted to correspond to the pig’s normal temperature. Hypothermia was prevented using warm blankets.

### Experimental procedures

**Fig. 1:**
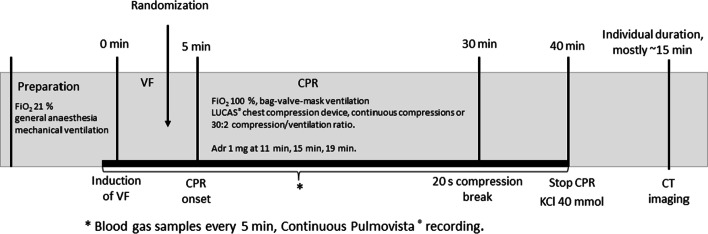
Timeline of the experimental protocol. Adr: adrenaline; FiO_2_: fraction of inhaled oxygen; VF: ventricular fibrillation; CPR: cardiopulmonary resuscitation; KCl: potassium chloride; CT: computed tomography

Figure 1 presents the timeline of the experiment. Prior to the induction of ventricular fibrillation (VF), baseline blood gas analysis was performed. VF was induced with a 9 V direct current. Once the cardiac arrest period began, mechanical ventilation and anaesthesia were stopped and the pacing wire was removed. The test subjects were randomized into two groups with sealed envelopes: continuous manual ventilation during compressions or a 30:2 ratio of compressions and ventilations. For practical reasons, the allocation was not blinded to the group performing the experiments. The compressions were started after a 5-min no-flow time, mimicking the delay in the arrival of the emergency medical service in the clinical setting. The compressions were performed mechanically (LUCAS 2 Chest Compression System, Jolife AB, a part of Stryker, Lund, Sweden). Manual bag-valve ventilation (LAERDAL Silicone Resuscitator, Laerdal Medical, Stavanger, Norway) was performed with supplemental 100% oxygen flow, either with a frequency of around 10–12/min in the CCC group or twice during the compression pause in the 30:2 group, targeting an approximate tidal volume of 500 ml in both groups. At 11-, 15-, and 19-min intervals from the start of VF (corresponding to two, four and six 2-min cycles of CPR after the 5-min no-flow time), a 1 mg bolus of adrenaline (epinephrine) was administered intravenously. At the 30-min time point, a 20-s break from compressions with continuous ventilation was taken to achieve a less disturbed EIT recording interval. Arterial blood gases were measured at 5-min intervals, and a blood sample from the central vein was taken at baseline and at 30 min from the arrest. At 40 min, the pigs were euthanized with a lethal 40 mmol dose of potassium chloride, and the intubation tube was clamped after insufflating the lungs with one full manual ventilation. The pigs were taken for a post-mortem CT lung scan to a CT scan unit, where the imaging was performed with the animals in the prone position.

### Cerebral oximetry

Cerebral oximetry was performed with near-infrared spectroscopy (NIRS) (INVOS TM5100C Cerebral Oximeter, Somanetics Inc., Troy, MI, USA). One sensor pad was attached to the right forehead and the other was attached as a control on the animal’s abdomen. We considered the recording values abnormal if the intra-arrest NIRS values rose to higher levels than those recorded before the onset of the cardiac arrest.

### Electrical impedance tomography

Ventilation distribution was assessed using electrical impedance tomography (EIT). The EIT belt was set around the chest just caudal to the mechanical compression device. The skin hair under the belt was shaved off, and the skin was cleaned with 70% ethanol to ensure the best possible contact. Because of the disturbance of the mechanical compressions, the four most medioventral sensors of the belt were replaced with Ambu WhiteSensor 4500 M-H electrodes, carefully aligned with the positions of the electrodes in the belt. The EIT data were recorded with Dräger Pulmovista® 500 (Drägerwerk AG & Co, Lübeck, Germany) and analysed with Dräger SW EITdiag V1.6 (Drägerwerk AG & Co, Lübeck, Germany).

**Fig. 2:**
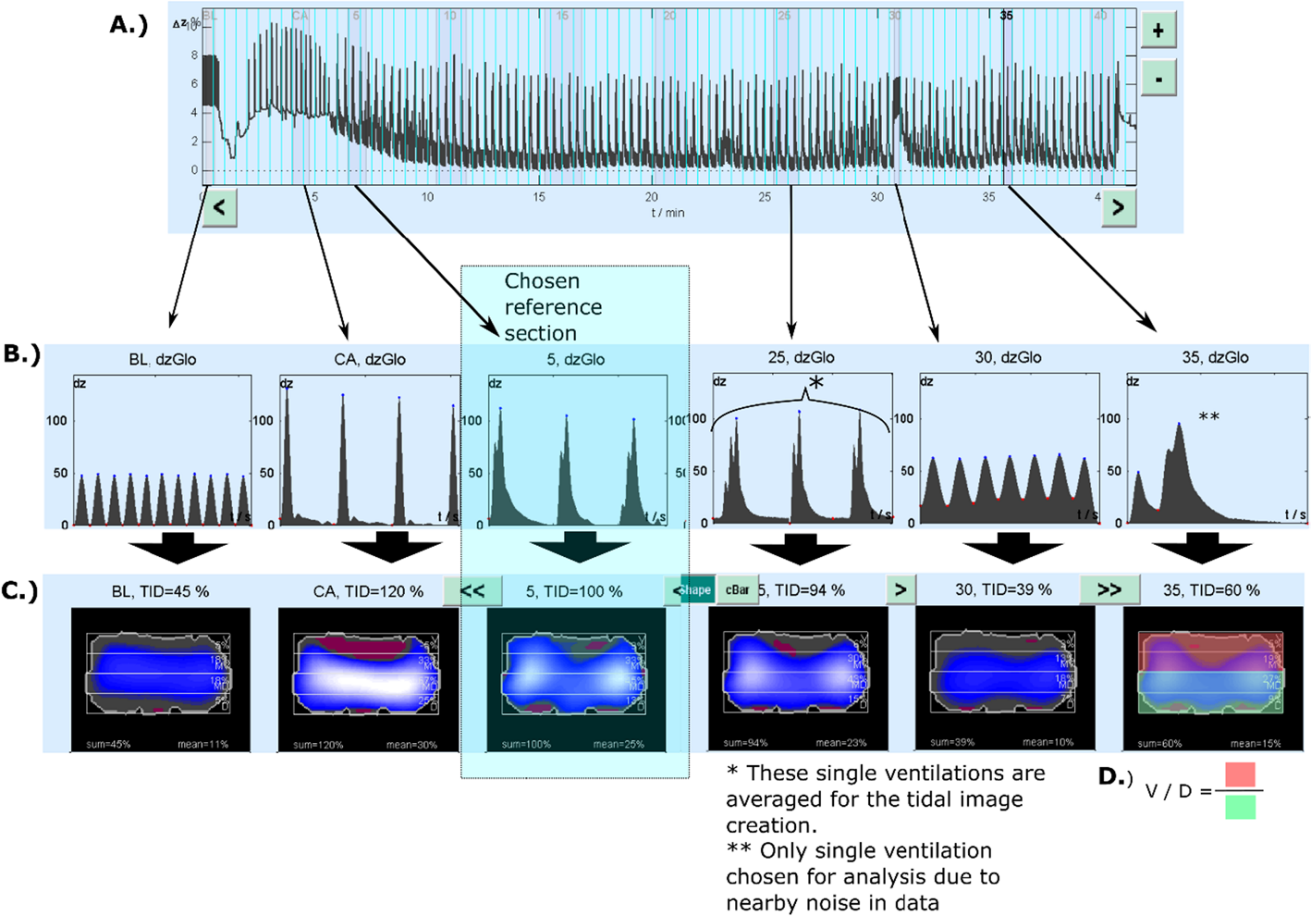
Schematic illustration of the EIT analysis work chart. **A** The raw dzGlo curve. **B** The reconstructed sections after filtering. **C** Visual slices created with the automated analysis scheme from filtered sections. **D** Visual explanation of the V/D index. BL: baseline; dzGlo: global impedance change; TID: tidal impedance change; CA: cardiac arrest; V/D: ventral per dorsal

An illustration of the EIT analysis is provided in Fig. 2. The raw recording was manually divided into sections of interest (baseline, cardiac arrest and 5-min intervals throughout the CPR). The reconstruction settings are provided in the Additional file 2. The reconstructed sections were analysed using automated methods provided by the analysis software to create visual slices and quantified data. We report the global tidal impedance change referenced to the 5-min (beginning of the CPR) section values and the ventral to dorsal (V/D) distribution indices computed as illustrated in Fig. 2. A V/D value of 1 means that the tidal changes in ventral and dorsal areas are equal and a value of 0 means that all tidal change happens dorsally.

### CT image analysis

The CT scans were evaluated by a veterinary radiologist (ML) blinded to the intervention group, and the volume measurements were performed with Osirix MD versions 12.0.3 and 12.5.0 (VetCT, Cambridge, UK). The HU values were measured from 10 lung slices chosen as described by Reske et al. [12]. The first slice was chosen from the most cranial aspect of the lungs, where both left and right cranial lobes are visible on the same transverse slice. The most caudal slice was chosen similarly where there was still enough lung on both hemithoraces for segmentation into 2–3 segments, but where the accessory lobe was no longer visible. Eight evenly spaced slices were chosen from between these slices.

The lung parenchyma was manually delineated separately for both hemithoraces and accessory lobes. The mean HU values, normalized to the corresponding surface area from these delineations, were used to calculate the mean HU of the overall lung. The hemithoraces were also divided into 2–3 ventrodorsal segments of equal height. Representative HU values from these segments were determined and averaged from all slices for the dorsal, middle and ventral HU values. Subjects with more than a mild pneumothorax were excluded from the analysis because of the dislocated anatomy.

### Statistical analysis and sample size

The variables are reported as medians and interquartile ranges (IQRs). The variables with single measurements were compared using the Mann–Whitney U test. The variables measured at multiple time points were compared with a linear mixed-effects model with unstructured symmetry as the covariance matrix. A diagonal covariance matrix was used with the NIRS data. The analysis included the effects of the interventional group, time and the interaction between time and the intervention group. The values over time were plotted as medians and IQRs. A multiple comparison test was also run as a sensitivity analysis. The sample size was estimated based on arterial blood gas data from Kim et al. [13]. According to this, a sample size of 30 divided into two equal groups would be sufficient to detect a 20 mmHg difference in PaO_2_ with a statistical power of 0.95. A p-value of less than 0.05 was considered significant.

## Results

### Pre-arrest state and markers of ventilation during CPR

All the randomized test subjects (30:2 group: *n* = 15, CCC group: *n* = 15) were included in the final analysis. The pigs were predominantly male, and the baseline characteristics of the groups were comparable (Table 1). Ventilation was performed similarly in both groups, with no difference in peak airway pressures or compliance between the groups during the CPR (Table 2). Within the CCC group, an average value of 10 ventilations per minute was achieved. The tidal volumes were significantly smaller in the 30:2 compared to the continuous group (*p* < 0.01).

**Table 1:** Baseline characteristics of the resuscitation groups

	30:2 group	CCC group	*p*-value
	Median (IQR)	Median (IQR)	
Weight (kg)	48 (46–49)	47 (47–50)	0.88
Heart rate (bpm)	125 (105–146)	133 (103–154)	0.46
FiO_2_ (%)	21 (21–21)	21 (21–21)	0.27
Peak pressure (cmH_2_O)	26 (24–29)	27 (24–28)	0.93
Tidal volume (ml)	354 (332–376)	346 (335–386)	0.94
Compliance (ml/cmH_2_O)	34 (30–37)	36 (33–37)	0.10
pH	7.6 (7.5–7.6)	7.6 (7.5–7.6)	0.97

IQR: interquartile range; CCC: continuous chest compressions; bpm: beats per minute; FiO_2_: fraction of inspired oxygen

**Table 2:** Vital signs and interventions during cardiopulmonary resuscitation

	30:2 group	CCC group	*p*-value
	Median (IQR)	Median (IQR)	
Ventilation rate (min^−1^)	6*	10 (9–12)	< 0.001
FiO_2_ (%)	95 (94–95)	95 (94–95)	0.90
Peak pressure (cmH_2_O)	39 (35–45)	39 (35–45)	0.90
Tidal volume (ml)	370 (290–420)	430 (360–480)	< 0.001
Compliance (ml/cmH_2_O)	19 (16–23)	19 (17–22)	0.95

CCC: continuous chest compressions; FiO_2_: fraction of inspired oxygen

*This is a calculated value based on the Lucas ® 2 30:2 protocol programming

### Oxygen, carbon dioxide, lactate and mean arterial pressure

**Fig. 3:**
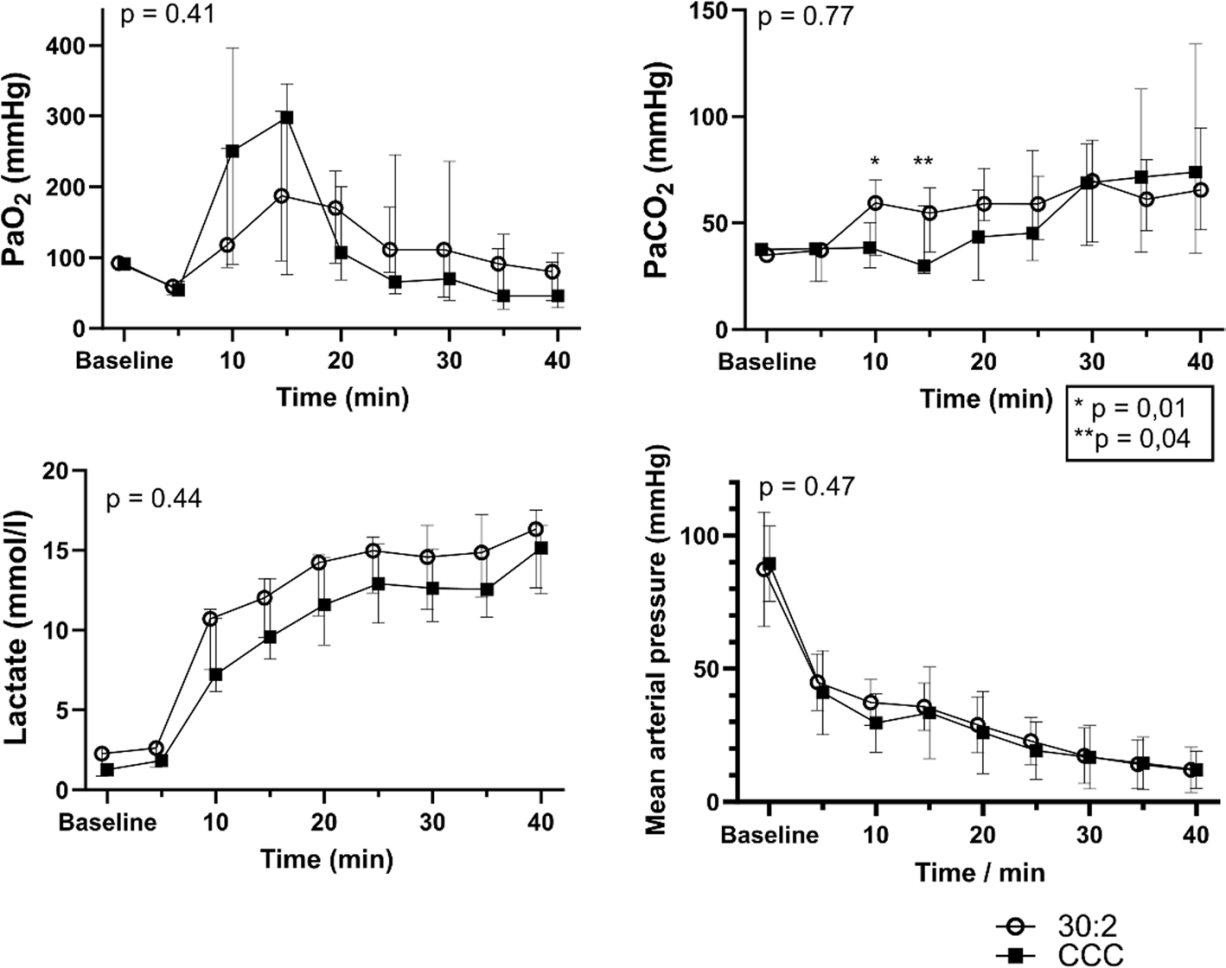
Arterial oxygen pressures (PaO_2_), carbon dioxide pressures (PaCO_2_), lactate levels and mean arterial pressures during experimental cardiopulmonary resuscitation shown as medians and interquartile ranges. The p-value is given for a linear mixed model between the groups. CCC: continuous chest compressions

The arterial blood oxygen, carbon dioxide, lactate and mean arterial pressure (MAP) results are shown in Fig. 3. There were no statistically significant differences between the groups for PaO2 (*p* = 0.41), PaCO2 (*p* = 0.77), lactate (*p* = 0.44), or MAP (*p* = 0.47) or in the time and group interaction terms in the PaO_2_ (*p* = 0.31), PaCO_2_ (*p* = 0.13), lactate (*p* = 0.13) or MAP levels (*p* = 0.15). However, the PaCO_2_ values at 10 min (*p* = 0.011) and 15 min (*p* = 0.041) were significantly higher in the 30:2 group when compared with the Mann–Whitney U test.

In a sensitivity analysis, where we excluded animals with a finding of a moderate-to-marked pneumothorax on the post-mortem CT scan, we found no difference in PaO_2_ levels over time between the two intervention groups (Figure in Additional file 3).

### End-tidal CO_2_

**Fig. 4:**
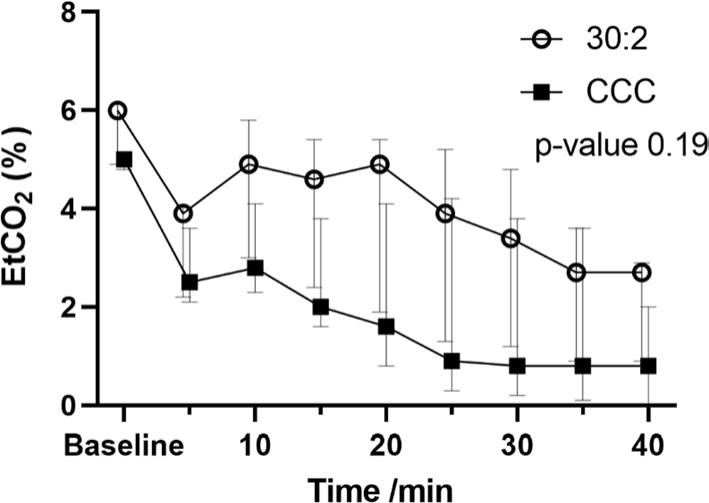
EtCO_2_ levels of the 30:2 and CCC groups during experimental cardiopulmonary resuscitation shown as medians and interquartile ranges. The p*-*value is given for a linear mixed model between the groups. EtCO_2_: end-tidal carbon dioxide; CCC: continuous chest compressions

The EtCO_2_ levels (Fig. 4) were similar between the groups (*p* = 0.19). The p-value for the group and time interaction was 0.048.

### NIRS

**Fig. 5:**
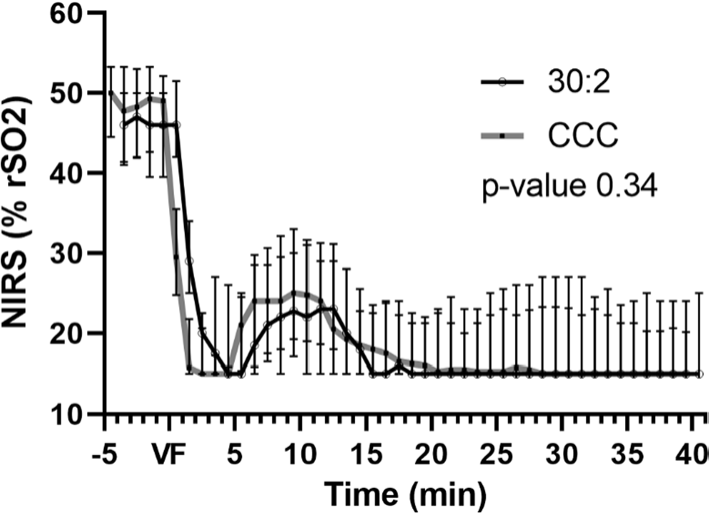
NIRS values during experimental cardiopulmonary resuscitation shown as medians and interquartile ranges. The p*-*value is given for a linear mixed model between the groups. NIRS: near-infrared spectroscopy; rSO_2_: regional oxygen saturation index; VF: ventricular fibrillation; CCC: continuous chest compressions

The cerebral NIRS values are presented in Fig. 5. One subject from the CCC group was eliminated from the analysis because of clearly abnormal measurement values suggesting technical failure in the measurement. There was no significant difference between the groups (*p* = 0.34) or between the time and group interaction terms (*p* = 1.00).

### EIT and CT

The main findings from the EIT data are reported in Table 3. There were no statistically significant differences between the groups in measured EIT parameters at 25 min and 30 min from the beginning of the CPR. In the 30:2 group, we saw recurring agonal breaths with the EIT recording in some subjects to persist even until the 30 min compression break.

**Table 3:** Electrical impedance tomography and computed tomography findings

EIT findings	30:2 group	CCC group	*p*-value
	Median (IQR)	Median (IQR)	
Tidal impedance change, BL (%)	52 (38–94)	53 (46–56)	0.75
Tidal volume distribution, BL	0.90 (0.67–0.96)	0.90 (0.74–1.01)	0.98
Tidal impedance change, 30 min (%)	123 (87–30)	99 (79–110)	0.1
Tidal volume distribution, 30 min	0.89 (0.75–1.10)	1.0 (0.69–1.15)	0.81
Tidal impedance change, 25 min (%)	94 (81–125)	96 (69–111)	0.35
Tidal volume distribution, 25 min	0.86 (0.78–0.99)	0.88 (0.76–1.03)	0.75
Tidal impedance change, 35 min (%)	103 (83–125)	87 (81–106)	0.22
Tidal volume distribution, 35 min	0.97 (0.85–1.06)	0.94 (0.78–1.13)	0.75
CT findings after death			
Total lung volume (ml)	1150 (880–1300)	1210 (890–1370)	0.65
Rib fractures (#)	5 (4–5)	5 (4–6)	0.68
Mean density (HU)	− 626 (− 553 to − 684)	− 575 (− 527 to − 729)	0.83
Ventral lung density (HU)	− 596 (− 503 to − 625)	− 547 (− 473 to − 658)	0.83
Middle lung density (HU)	− 711 (− 619 to − 798)	− 730 (− 690 to − 801)	0.53
Dorsal lung density (HU)	− 741 (− 717 to − 835)	− 809 (− 756 to − 856)	0.24

The values are reported as medians and interquartile ranges. The tidal volume distributions were calculated by dividing the impedance change in the ventral region of interest by the impedance change in the dorsal region of interest. The 5-min recording section was used as a reference section, and impedance changes are reported as comparisons to the 5-min tidal impedance change. EIT: electrical impedance tomography; CCC: continuous chest compressions; BL: baseline

The main CT findings are reported in Table 3. There were no statistically significant differences between the groups in total lung volume, the number of rib fractures, the severity of the dislocation of the thoracic cage, the mean HU value of the lungs or the dorsal, middle and ventral HU values. There was a trend of lower HU values towards the dorsal lung region, as expected. We encountered 11 animals with pneumothoraces. The 30:2 group included 3 minimal to mild and 3 moderate pneumothoraces. In the CCC group, there were 1 minimal to mild, 1 moderate and 3 marked pneumothoraces. The proportion of pneumothoraces was compared with Fisher’s exact test, and there was no statistically significant difference between the groups. The moderate-to-marked pneumothorax subjects were excluded from the HU value analyses, leaving 12 subjects in the 30:2 group and 11 subjects in the CCC group.

## Discussion

### Key findings

Contrary to our primary hypothesis, we found no difference in this experimental model between a 30:2 or a continuous compression-to-ventilation ratio with regard to intra-arrest levels of arterial oxygen, carbon dioxide or lactate. There seemed to be a faster deterioration of EtCO_2_ values in the CCC group compared to the 30:2 protocol. Also contrary to our hypotheses, there were no differences between the groups with regard to EIT data and post-mortem CT findings, suggesting no major difference in lung injury with these compression-to-ventilation protocols.

### Relationship with previous studies

Safar et al., in their early CPR works, already showed that CCC alone is not sufficient to produce adequate ventilation during CPR [14], and this has also been confirmed for mechanical compressions [15]. The importance of adequate ventilation is underlined with refractory CA, and the emergence of ECPR creates pressure to optimize the treatment of this patient group. As intra-arrest oxygen levels may be important regarding even long-term neurological outcome, the optimal ventilation strategy during prolonged CPR needs to be defined.

Yang et al. [16] compared the 30:2 ratio to CCC during a shorter period of CPR. All their animals achieved a return of spontaneous circulation (ROSC), and the haemodynamic and ventilatory parameters were comparable between the groups. However, there were more rib fractures in the CCC group in their study. Other prior research with animals has mainly focused on comparing shorter compression periods between ventilations, such as 15:1 and 15:2 [17] and compressions only in 30:1 and 30:2 protocols [13]. The longer compression phase seems to provide better haemodynamic parameters without major compromises in blood gas values. Clear differences in ROSC rates have not been demonstrated, however.

Three mechanisms have been proposed as contributing to aggravating hypoxia during prolonged CPR. First, the formation of dynamic atelectasis due to compressions and a progressive reduction in the lung volume with no or insufficient ventilation creates a ventilation perfusion mismatch and a reduction of the functional lung volume [18, 19]. Second, intrathoracic airway closure connected to intrathoracic pressure swings caused by chest compressions may inhibit adequate ventilation [5, 6, 20]. Finally, the possible development of CRALE was recently shown by Magliocca et al. [10]. The waveform capnogram-derived airway opening index (AOI) has been used to quantify airway closure and has been associated with CRALE, which itself seems aggravated by mechanical compression compared to manual compression [10, 11]. These findings support the hypothesis that mechanical CCC has a negative effect on gas exchange. Deleterious effects of hyperventilation have also been demonstrated [21, 22], which limits options for upregulating the volume or frequency of continuous ventilation.

Adding positive end-expiratory pressure (PEEP) to CCC and continuous ventilation has been proposed to counter airway closure [23], but this intervention lacks the patient data available for the 30:2 protocol. Successful animal studies on chest compression synchronized ventilation (CCSV) have been conducted [24, 25], but this protocol also lacks clinical data from patients.

According to our findings, a continuous or 30:2 compression-to-ventilation ratio results in similar blood gas exchange during prolonged CPR. There are a few possible reasons for this. It is possible that the atelectatic lung areas formed during compressions do not resolve well enough during the compression pauses for ventilation. These lung areas might require low continuous PEEP during compressions to remain open. The more slowly deteriorating trend of EtCO_2_ with higher PaCO_2_ values in the 30:2 group could have been caused by insufficient overall ventilation in this group.

The trend in the PaO_2_ curves displays an early efficient but quite soon deteriorating trend in arterial blood oxygenation for the CCC group compared to the flatter but more sustained curve of the 30:2 group. The deterioration seemed to coincide with a more rapid decrease in perfusion pressures that happened over time despite the ongoing mechanical CPR. It could also be due to some form of evolving pathology, such as a hyperinflation-induced rise in intrathoracic pressure leading to compromised return of blood to the heart and aggravated ventilation perfusion mismatch. Future interest lies in studying whether adding PEEP to ventilation support during CPR would provide better ventilatory outcomes without compromising circulation.

### Electrical impedance tomography

EIT technology allows bedside, real-time, dynamic assessment of lung function, especially the distribution of ventilation. The technology provides a spatialized profile of tidal lung aeration with possibilities to identify not only areas of atelectasis and non-aeration but also hyperinflation and overdistention [26]. Because spatial resolution is on a scale of chest segments of 7–10 cm, the accumulating information is evaluated in regions of interest (ROI) using a layered or quadrant segmentation strategy. The images generated always relate to a chosen reference section within the recording, since absolute impedance values fluctuate greatly. The impedance variations of perfusing the blood and the heart function are filtered out using a low-pass post-processing filter, which also seems to filter out most of the chest compression-related variation from the raw dzGlo (global change of impedance curve). To the best of our knowledge, this technology has been applied to CPR settings only once prior to our study [19]. Hartman et al. compared the EIT data from baseline measurements to measurements during CPR, reporting a dorsal-to-ventral shift in the impedance changes. We do not report such a shift. The analysis of EIT data interpretation lost sensitivity in spatial discrimination and tidal volumes due to manual ventilation during CPR. Some pigs also maintained small volume spontaneous breathing until the 30-min time point, which we subtracted from the EIT analysis by analysing a single ventilation instead of a mean of three or more ventilations. Due to these data quality limitations, we decided to report only robust indices of the distribution of ventilation and leave out, for example, global inhomogeneity indices and 4/1 ROI values. The V/D values showed slightly dorsally emphasized tidal ventilation, possibly representing hyperinflation with reduced tidal ventilation of the ventral lung regions. This is especially interesting, since the post-mortem CTs showed heavily dorso-caudally allocated non-ventilating lung tissue, which seemed not to redistribute the ventilation in the EIT recording. As can be witnessed from Fig. 2, the mechanical ventilation and agonal breathing distributions differed from the intra-arrest distributions.

### Computed tomography

The current study suggests that even though injuries are common in experimental CPR, there is no difference in the 30:2 compression-to-ventilation ratio compared to continuous compressions. Several clinical studies have examined skeletal, cardiac and abdominal injuries related to CPR [27–34]. Overall, injuries appear more frequent with the use of mechanical compression devices, longer CPR times and older age. The prevalence of lung injury has been reported to range from 4 to 15% [28, 33]. Cha et al. [35] published a study of 91 patients with chest CT scans taken within 24 h of ROSC and showed a high proportion (41%) of lung contusions affecting oxygenation and predisposing to acute respiratory distress syndrome (ARDS) but no association with mortality or neurological outcome. Similar findings have been presented by other groups [10]. In our study, rib fractures were common, which compares with human studies reporting rates of between 50 and 80% [28]. The median overall lung HU values remained above -500 HU, implying that more of the lung tissue remained aerated than non-aerated through our experiment in both groups.

### Study limitations

A porcine experimental model of CPR was used because the size of the chest and the heart of a pig resembles that of humans. However, the chest of a pig is more barrel-shaped than the human chest, which might prevent airway closure phenomenon, thus impairing the generalizability of the results to human CPR. We decided to report the calculated and not measured ventilation frequency value for the 30:2 protocol, because of interference caused by agonal breathing by some of the subjects and partly by the chest compression induced EtCO2 oscillations. The same problem was not experienced in a substantial magnitude in the more continuously ventilated CCC group.

Further on, peak airway pressures between the study groups did not differ in our experiment. Ventilations were provided by a member of the team in a standardized fashion and it is possible that in case of increased resistance the resuscitator used less force. On the other hand, we think that our setting corresponds to the clinical situation. The lower tidal ventilatory volumes in the 30:2 group are partly explained by the short 3-s break provided by the Lucas ® 2 to perform the two sequential ventilations.

With EIT data processing, we selected a filter of 30 bpm instead of the more conventional 60 bpm to clear the chest compression oscillation more effectively out of the dzGlo curves. This resulted in the 30:2 protocol’s sequential ventilations fusing partly together and being analysed as one. This did not affect the maximal impedance delta value of those two ventilations and probably affected only little on the distribution of ventilation, since the sequential ventilations tended to distribute similarly**.**

### Conclusions

Our study suggests no difference in arterial blood levels of oxygen, carbon dioxide and lactate with compression-to-ventilation ratios of 30:2 compared to CCC with prolonged mechanical CPR. The distribution pattern of ventilation during the 25–35 min phase of CPR was similar measured with EIT as were the post-mortem CT findings of the prevalence and type of lung injury. We report a marked deterioration in arterial oxygen levels, EtCO_2_ levels and MAP in both intervention groups.

## Supplementary Information

**Additional file 1.** The Arrive checklist for the manuscript.

**Additional file 2.** The Electrical impedance tomography reconstruction settings used for filtering the raw curves.

**Additional file 3.** The PaO2 levels during experimental cardiopulmonary resuscitation shown as medians and interquartile ranges. The p-value is given for a linear mixed model between the groups. The subjects with pneumothoraces are excluded in these graphs.

## Data Availability

The datasets used and/or analysed during the current study are available from the corresponding author on reasonable request.

## Abbreviations

CCC: Continuous chest compressions
CPR: Cardiopulmonary resuscitation
CRALE: Cardiopulmonary resuscitation-associated lung oedema
CT: Computed tomography
ECPR: Extracorporeal cardiopulmonary resuscitation
EIT: Electrical impedance tomography
EtCO_2_: End-tidal carbon dioxide
FiO_2_: Fraction of inspired oxygen
NIRS: Near-infrared spectroscopy
PaCO_2_: Partial pressure of carbon dioxide
PaO_2_: Partial pressure of oxygen
ROSC: Return of spontaneous circulation
SpO_2_: Oxygen saturation
V/D: Ventral to dorsal
VF: Ventricular fibrillation
