# Supplementary figures and images for: Ventilation during continuous compressions or at 30:2 compression-to-ventilation ratio results in similar arterial oxygen and carbon dioxide levels in an experimental model of prolonged cardiac arrest
Source: Intensive Care Med Exp. 2023 Jan 6;11:3. doi: 10.1186/s40635-022-00485-0 (PMC9823175; doi:10.1186/s40635-022-00485-0)

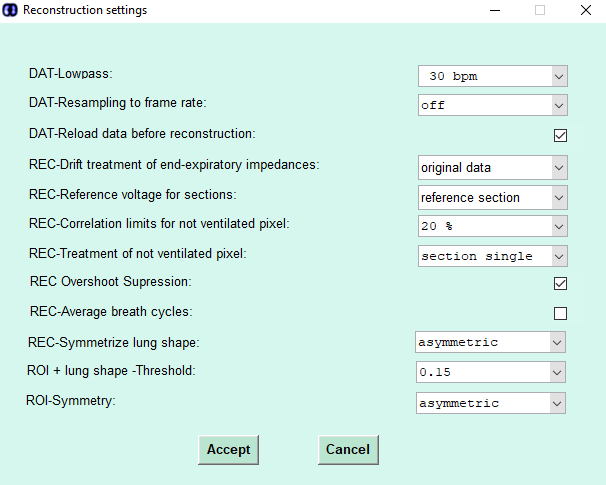

Supplement: Supplementary file 2 — Additional file 2. The Electrical impedance tomography reconstruction settings used for filtering the raw curves. [file 40635_2022_485_MOESM2_ESM.tiff]

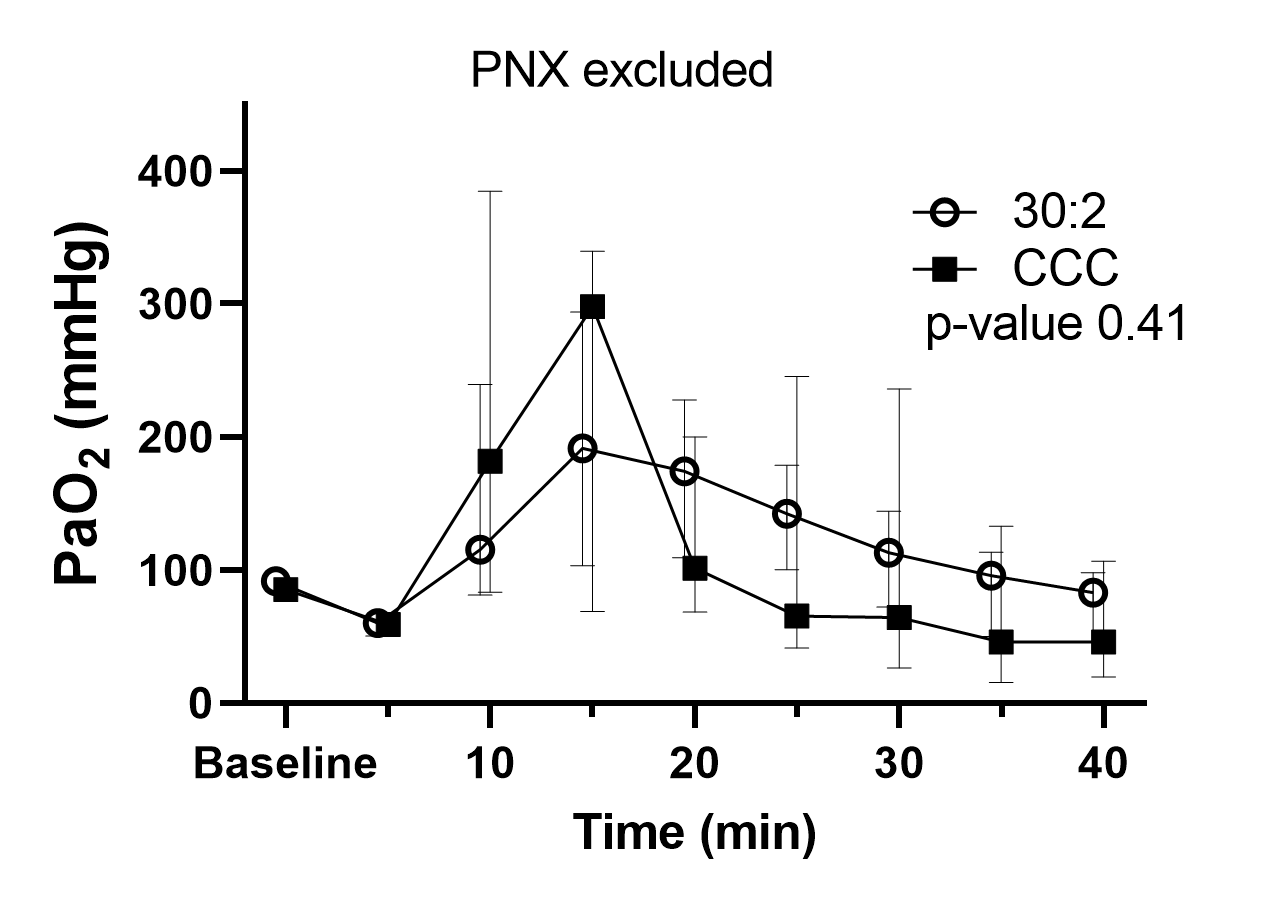

Supplement: Supplementary file 3 — Additional file 3. The PaO2 levels during experimental cardiopulmonary resuscitation shown as medians and interquartile ranges. The p-value is given for a linear mixed model between the groups. The subjects with pneumothoraces are excluded in these graphs. [file 40635_2022_485_MOESM3_ESM.tif]
